# Population dynamics of migrant wheat aphids in China’s main wheat production region and their interactions with bacterial symbionts

**DOI:** 10.3389/fpls.2023.1103236

**Published:** 2023-02-09

**Authors:** Tong Li, Gongqiang Yang, Qian Li, Yueli Jiang, Dongmei Kang, Zhiye Fan, Zhongjun Gong, Ruijie Lu, Guotao Zhou, Yuqing Wu, Chuantao Lu

**Affiliations:** ^1^ Institute of Plant Protection/Henan Key Laboratory of Crop Pest Control/Key Laboratory of Integrated Pest Management on Crops in Southern Region of North China, Henan Academy of Agricultural Sciences, Zhengzhou, China; ^2^ College of Bioscience and Resource Environment/Key Laboratory of Urban Agriculture (North China), Ministry of Agriculture and Rural Affairs of the People’s Republic of China, Beijing University of Agriculture, Beijing, China; ^3^ Institute of Plant Protection, Luohe Institute of Agricultural Sciences, Luohe, China; ^4^ Henan Yunfei Technology Development Co., Ltd, Zhengzhou, China

**Keywords:** wheat aphid, aphid symbionts, insect migration, *Sitobion miscanthi*, *Rhopalosiphum padi*, *Schizaphis graminum*

## Abstract

*Sitobion miscanthi*, *Rhopalosiphum padi*, and *Schizaphis graminum* are the three main pests in Chinese wheat-producing regions. In 2020, they are classified into the Chinese Class I list of agricultural diseases and pests, due to their severe harm to wheat plantings. *S. miscanthi*, *R. padi*, and *S. graminum* are migrant pests, and understanding their migration patterns and simulating their migration trajectories would improve forecasting and controlling them. Furthermore, the bacterial community of the migrant wheat aphid is also less known. In this study, we employed a suction trap to uncover the migration patterns of the three wheat aphid species in Yuanyang county, Henan province, during 2018 to 2020. And then the migration trajectories of *S. miscanthi* and *R. padi* were simulated using the NOAA HYSPLIT model. The interactions between wheat aphids and bacteria were further revealed by specific PCR and 16S rRNA amplicon sequencing. The results showed that the population dynamics of migrant wheat aphids was varied. Most of the trapped samples were identified to be *R. padi*, and *S. graminum* was the least collected sample. Typically, *R. padi* had two migration peaks in the 3 years, whereas *S. miscanthi* and *S. graminum* only exhibited one migration peak in 2018 and 2019. Moreover, the aphid migration trajectories varied over the years. Generally, the aphids originated from the south and migrated to the north. Herein, the infections of three main aphid facultative bacterial symbionts, *Serratia symbiotica*, *Hamiltonella defensa*, and *Regiella insercticola*, were detected in *S. miscanthi* and *R. padi* with specific PCR. *Rickettsiella*, *Arsenophonus*, *Rickettsia*, and *Wolbachia* were further identified with 16S rRNA amplicon sequencing. Biomarker searching indicated that *Arsenophonus* was significantly enriched in *R. padi*. Furthermore, diversity analyses showed that the bacterial community of *R. padi* had a higher richness and evenness than that of *S. miscanthi*. In conclusion, this study expands our knowledge about the migration patterns of aphids in the main wheat plant region of China and reveals the interactions between bacterial symbionts and migrant aphids.

## Introduction

Wheat (*Triticum aestivum* L.) is the third major grain crop plant in China. The most common pest on wheat is the wheat aphid, which not only feeds on the grain but also spreads plant viruses like the barley yellow dwarf virus (BYDV). Studies have reported that approximately 10–15 million hectares of wheat fields in China are affected by aphid infestations, resulting in 10% yield losses annually (Hu et al., 2016; Gong et al., 2021). Indian grain aphid *Sitobion miscanthi*, Bird cherry-oat aphid *Rhopalosiphum padi* (Linnaeus), and greenbug *Schizaphis graminum* are three main wheat aphids in China. In 2020, due to their severe harm to wheat plantings, the three wheat aphids are classified into the Chinese Class I list of agricultural diseases and pests (http://www.zzys.moa.gov.cn/gzdt/202006/t20200604_6345940.htm). It is well known that wheat aphids can perform long-distance migration by air (Sun et al., 2022); hence, uncovering their seasonal migration patterns would improve forecasting and controlling them.

The insect marking–release–recapture technique (Hagler and Jackson, 2001) and trapping aerial insect samples using the sticky nets attached to the balloons and airplanes (Florio et al., 2020) have been used to track the migration of insects, but these strategies are labor-intensive. To date, the economical searchlight trap and high effective radar are widely employed to investigate insect migrations. However, the searchlight trap is mostly used to uncover the migrations of lepidopterous pests with large bodies (Guo et al., 2020; Zhou et al., 2021; Guo et al., 2022; Wang et al., 2022b), and the commonly used centimeter-wave vertical-looking radar is unsuitable for monitoring small insects, such as aphids, and a more expensive radar with a shorter wavelength is required (Chapman et al., 2003; Chapman et al., 2004; Feng et al., 2009). Suction traps have been used to monitor the aerial movement of aphids since 1964 (Macaulay et al., 1988), which probably filled the gap in the investigation of aphid migrations. Suction trap networks have been established in the United Kingdom and the United States to monitor seasonal distribution and abundance of diverse aphid species for a few decades (Bell et al., 2015; Lagos-Kutz et al., 2020). In Spain, suction traps are useful for monitoring the flight of damson-hop aphid (*Phorodon humuli*) at the start of spring (Pérez et al., 2006). In 2011, to investigate the migrations of soybean and wheat aphids, a Chinese aphid suction trap network was constructed (Qiao et al., 2011).

It is well known that aphids and bacteria share intimate relationships. In recent years, the 16S ribosomal RNA (rRNA) gene amplicon high-throughput sequencing has been applied to explore the aphid bacterial community (Xu et al., 2020; Xu et al., 2021a; Xu et al., 2021b). However, these bacterial surveys have mainly been performed in the field-collected samples; the interactions between migrant aphids and bacterial symbionts are less studied. In this study, which employed a suction trap, we uncovered the occurrence and migration patterns of the three main wheat aphids in Yuanyang county from 2018 to 2020 and simulated their migration trajectories. Furthermore, using specific PCR and 16S amplicon sequencing, we uncovered the bacterial composition in the migrant *S. miscanthi* and *R. padi*.

## Materials and methods

### Flying wheat aphid collection and identification

Henan province in China, which contributes a quarter of China’s annual wheat harvest, is the core wheat production area. Notably, the first suction trap in Henan was constructed in Yuanyang county, Xinxiang city (35.01 N, 113.69 E). The suction trap is 8.8 m tall and collected the tiny flying insects weekly from April to June 2018–2020. Morphological characteristics were used to identify the three main wheat aphids, *S. miscanthi*, *R. padi*, and *S. graminum*, among the trapped samples. All samples were deposited in the Institute of Plant Protection, Henan Academy of Agricultural Sciences, Zhengzhou, China. They were stored in 75% ethanol and maintained under −20°C until DNA extraction.

### Wheat aphid migration pattern and trajectory analysis

The population dynamics of migration wheat aphids was summarized, and their migrating peaks were revealed. The HYSPLIT (HYbrid Single-Particle Lagrangian Integrated Trajectory) model is designed to simulate the trajectory of substances transported and dispersed through atmosphere using gridded meteorological data (Stein et al., 2015; Rolph et al., 2017). It is widely used to simulate the global dust distribution, volcanic ash dispersion, and pollutant transport (Stein et al., 2007; Stunder et al., 2007; Wang et al., 2011). Tiny insects like aphids have limited flight capacity. The flight speed of aphids is approximately 0.9 m s^−1^ (Robert, 1987), and the aphid flights are controlled by the wind when they move above at approximately 1 m from the ground (Parry, 2013). Hence, the HYSPLIT model is also suitable to calculate the migration trajectory of tiny insects with meteorological data.

In this study, the migration trajectories of *S. miscanthi* and *R. padi* were simulated with the online HYSPLIT model (https://www.ready.noaa.gov/HYSPLIT_traj.php). In these analyses, the meteorological data were obtained with the one-degree GDAS (Global Data Assimilation System) model, the model calculated star times were set on the 02:00 UTC time (10 a.m. in Beijing time) on the aphid migration peak days, and the flight heights were chosen as 10, 50, and 100 m AGL (above ground level). The aphid migration trajectories were simulated in 24 h.

### DNA extraction, bacterial symbiont detections, and phylogenetic analysis

In this study, the infections of three main aphid bacterial symbionts, *Serratia symbiotica*, *Hamiltonella defensa*, *Regiella insercticola*, and one common insect bacterial symbiont, *Wolbachia*, were detected in *S. miscanthi* and *R. padi* with specific primers listed in **Table S1**. In these specific amplifications, aphid total DNA was extracted from a single aphid using an Ezup Column Animal Genomic DNA Purification Kit (Sangon Biotech, Shanghai), following the manufacturer’s recommendations. Before DNA extraction, every aphid was washed with 70% ethanol and sterile water several times to remove surface contamination. Aphid *elongation factor-1α* gene was used as a reference to evaluate the extracted DNA quality, and the low-quality ones were excluded in the bacterial symbiont detections. The 16S rRNA sequences of the known aphid bacterial symbiont strains were retrieved to uncover the phylogenetic positions of the identified bacterial symbiont strains of *S. miscanthi* and *R. padi*. The sequences were aligned using the MUSCLE program in MEGA 7.0 (Kumar et al., 2016). Phylogenetic analysis was performed in IQ-TREE 1.6.12 (Nguyen et al., 2015). The support for each node was assessed by resampling 5,000 ultrafast bootstraps (Hoang et al., 2018). The best substitution model was selected by Bayesian information criterion in ModelFinder (Kalyaanamoorthy et al., 2017). The phylogenetic tree was visualized in Figtree 1.4.2 (http://tree.bio.ed.ac.uk/software/figtree/).

### Bacterial 16S rRNA amplicon amplification and sequencing

Herein, the bacterial composition of *S. miscanthi* and *R. padi* was further uncovered by amplicon sequencing. We randomly selected 15 individuals of *S. miscanthi* and *R. padi* collected in the same year and divided them into five replicates. The genomic DNA of the pooled aphids in each replicate was extracted using the DNeasy Blood and Tissue Kit (QIAGEN, Germany) according to the manufacturer’s instructions. The possible surface contamination was removed as described above. An approximately 460-bp fragment of the bacterial 16S rRNA V3–V4 region was amplified with primers 341F: CCTACGGGNGGCWGCAG and 806R: GGACTACHVGGGTATCTAAT (Qin et al., 2021). The PCR reactions were performed in triplicate in a 50-μl mixture containing 5 μl of 10 × KOD Buffer, 5 μl of 2 mM dNTPs, 3 μl of 25 mM MgSO_4_, 1.5 μl of each primer (10 μM), 1 μl of KOD Polymerase, and 100 ng of template DNA. PCR amplifications were carried out with the following program: 94°C for 2 min, followed by 30 cycles at 98°C for 10 s, 62°C for 30 s, and 68°C for 30 s and a final extension at 68°C for 5 min. The amplicons were further purified using the AxyPrep DNA Gel Extraction Kit (Axygen Biosciences, Union City, CA, USA) according to the manufacturer’s instructions. Subsequently, the purified amplicons were quantified using the ABI StepOnePlus Real-Time PCR System (Life Technologies, Foster City, USA), pooled in equimolar and generated the sequencing libraries. The next sequencing was performed by the Gene Denovo Biotechnology Co. (Guangzhou, China) on an Illumina HiSeq 2500 platform with a 2×250-bp paired-end method according to the standard protocols.

### Bioinformatics analyses

The reads were trimmed using Btrim with the cutoff for average quality scores higher than 20 over a 5-bp window size and a minimum length of 180 bp (Kong, 2011). The reads ranging from 242 bp to 244 bp were employed in the following analyses with QIIME2 (version 2022.11) (Bolyen et al., 2019). Primers and adaptor sequences were removed by cutadapt plugin (Martin, 2011). ASVs (amplicon sequence variants) were obtained by merging the sequences and removing chimera with the DADA2 plugin (Callahan et al., 2016). The taxonomic assignment of representative sequences was carried out using the feature-classifier plugin against the SILVA database (silva-138-99). Four alpha diversity indexes, ACE (abundance-based coverage estimator), Chao1, Shannon, and Simpson, were calculated in QIIME2; Bray–Curtis distance matrix was calculated in QIIME2, and then PCoA (principal coordinates analysis) and NMDS (non-metric multi-dimensional scaling) were visualized with Bray–Curtis distances and plotted in the ggplot2 package. The relative abundance of the top 10 bacterial taxa among the samples and the cladogram of the differential species were analyzed and visualized by the MicrobiotaProcess package (https://github.com/YuLab-SMU/MicrobiotaProcess), with the feature table and taxonomy table obtained in QIIME2.

## Results

### Annual migration patterns of three wheat aphids

**Figure 1:**
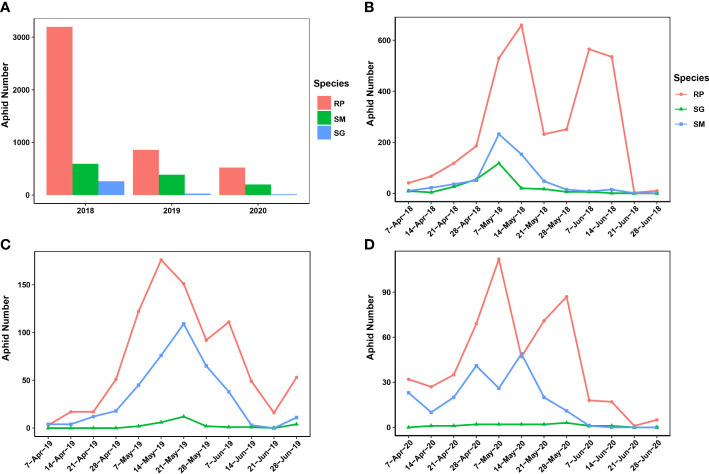
Population dynamics and migration patterns of the wheat aphids uncovered by a suction trap in Yuanyang country from 2018 to 2020. **(A)** The population dynamics of the three wheat aphids during 2018–2020. **(B–D)** The migration pattern of the three wheat aphids was revealed during 2018–2020. SM, *Sitobion miscanthi*; RR, *Rhopalosiphum padi*; SG, *Schizaphis graminum*.

In this study, most of the migrant wheat aphids were trapped in 2018 (4,051 samples), and 2020 had the least number of trapped migrant wheat aphids (737 samples). In 2019, 1,271 migrant wheat aphids were trapped (**Figure 1A**). *R. padi* was predominant among the samples of trapped wheat aphids, whereas *S. miscanthi* and *S. graminum* were less common. However, among the three aphid species, the number of trapped aphids was not significantly divergent (ANOVA test, *F* = 2.334, *p* = 0.1778). In addition, different patterns of wheat aphid migration were discovered over the years (**Figures 1B–D**). The results indicated that the migrations of *R. padi* had two distinct peaks during 2018–2020. Specifically, in 2018 and 2019, the first *R. padi* migration peak occurred in the second week of May, and the second peak occurred in the first week of June. However, in 2020, the two *R. padi* migration peaks happened 1 week earlier than that of 2018 and 2019. In contrast, only one peak was observed in the migration of *S. graminum* and *S. miscanthi* in 2018 and 2019, which occurred in the first and third weeks of May, respectively. The *S. miscanthi* migration experienced two adjacent peaks in 2020, which occurred in the fourth week of April and the second week of May, respectively. However, no obvious peak was revealed in *S. graminum* migration in 2020, since at most three samples were trapped in the weeks and a total of 15 samples were collected.

**Figure 2:**
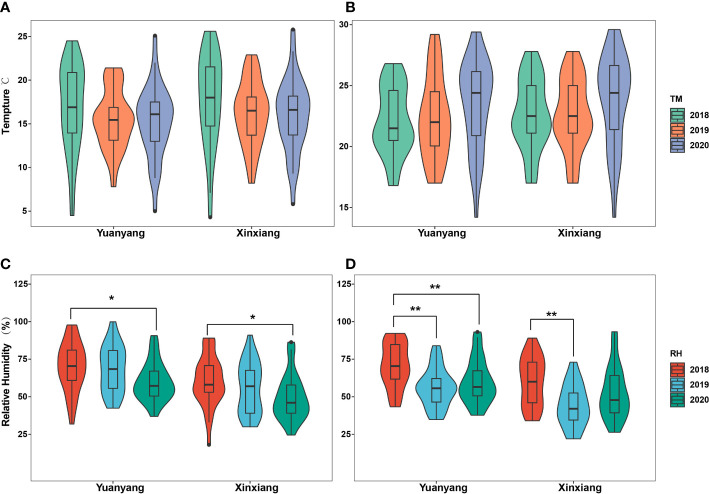
Statistical analysis of temperature and relative humidity among years in Yuanyang and Xinxiang regions. **(A, B)** Analysis with the April and May temperature obtained in Yuanyang and Xinxiang regions, respectively. **(C, D)** Analysis with the April and May relative humidity in Yuanyang and Xinxiang regions, respectively. *, indicating significant (p<0.05); **, indicating very significant (p<0.01).

Here, the meteorological data reflecting the local climate conditions near the suction trap (thereafter named Yuangyang), as well as those reflecting the climate conditions of a wider area, such as Xinxiang city, were involved in the statistical analyses to comprehensively uncover the underlying roles of temperature and humidity on the migrations of wheat aphids. The results indicated that there was no significant difference in the temperatures of April and May during 2018–2020, in either the Yuanyang or Xinxiang region (ANOVA test, *p* > 0.05) (**Figures 2A, B**). However, a significant difference in their relative humidity was observed (ANOVA test, *p* < 0.05) (**Figures 2C, D**). Furthermore, the multiple comparisons using Tukey’s test revealed that the relative humidity of April 2018 was significantly different with that of 2020 either in the Yuanyang or Xinxiang region. However, the relative humidity of May 2018 was significantly different from that of 2019 and 2020 in the Yuanyang region, and in the Xinxiang region, the relative humidity of May 2018 was significantly different from that of 2019.

### Aphid migration trajectory analyses

**Figure 3:**
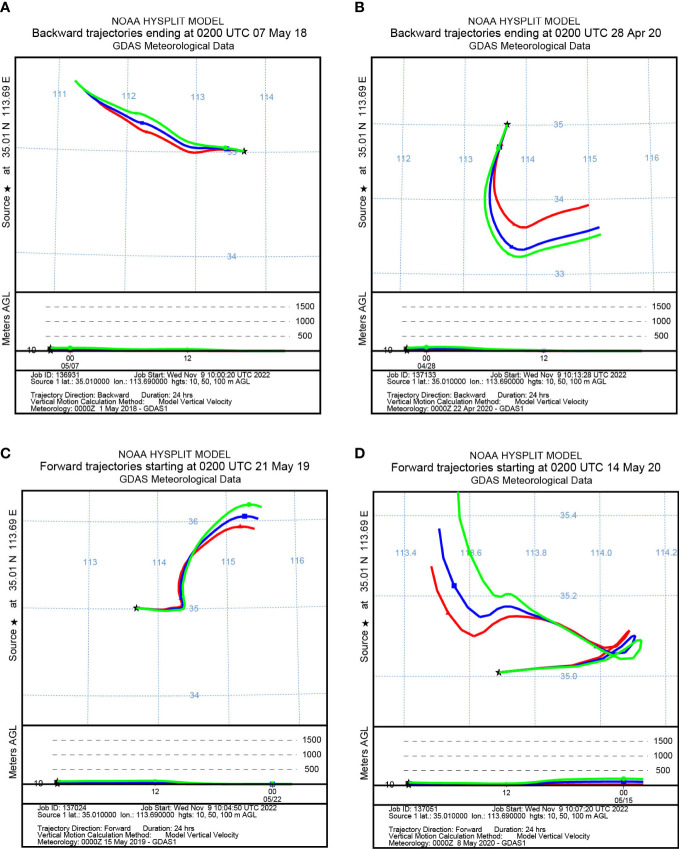
The migration trajectories of *Sitobion miscanthi* simulated by HYSPLIT. **(A, B)** Simulation of the backward trajectories on 7 May 2018 and 28 April 2020, respectively. **(C, D)** Simulation of the forward trajectories on 21 May 2019 and 14 May 2020, respectively.

**Figure 4:**
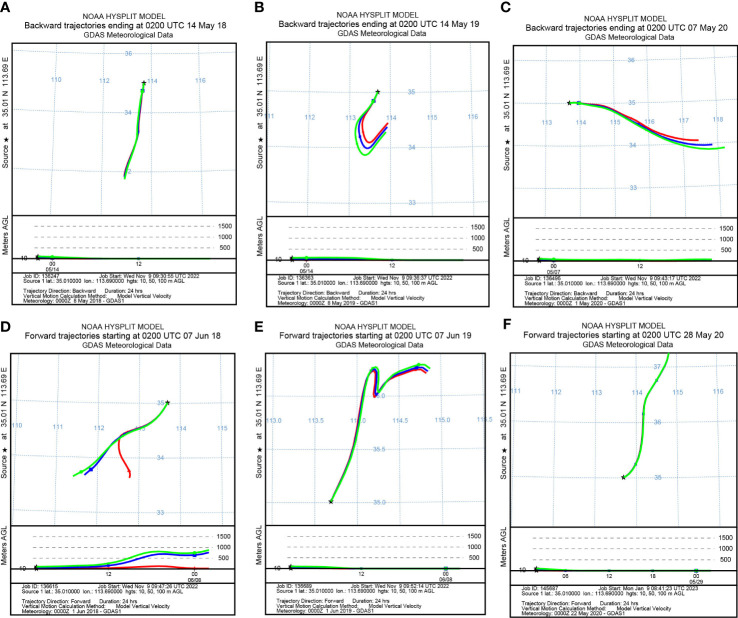
The migration trajectories of *Rhopalosiphum padi* simulated by HYSPLIT. **(A, B)** Simulation of the backward trajectories on 14 May 2018 and 14 May 2019, respectively. **(C)** Simulation of the backward trajectories on 7 May 2020. **(D, E)** Simulation of the forward trajectories on 7 June 2018 and 7 June 2019, respectively. **(F)** Simulation of the forward trajectories on 28 May 2020.

To identify the origin of the migrating aphids, we simulated the backward migration trajectories of immigrations. On the other hand, the forward migration trajectories of emigrations were simulated to uncover the destinations of the flying aphids. The results demonstrated similar migration trajectories at the three layers: 10 m, 50 m, and 100 m AGL (**Figures 3**, **4**). Most *S. miscanthi* migrations on 7 May 2018 and 28 April 2020 originated from the west and southeast of Yuanyang county, respectively (**Figures 3A, B**). In addition, on 21 May 2019 and 14 May 2020, *S. miscanthi* migrated to the northeast and northwest of Yuanyang county, respectively (**Figures 3C, D**). The migration trajectories of *R. padi* are summarized in **Figure 4**. The results indicated that most *R. padi* migrations on 14 May 2018 originated north of Yuanyang country (**Figure 4A**). However, most of the migrating *R. padi* on 14 May 2019 and 7 May 2020 originated northeast of Yuanyang country (**Figures 4B, C**). According to the simulation of forward migration trajectories, most *R. padi* would migrate to the south and east of Yuanyang county on 7 June 2018 (**Figure 4D**). On the contrary, most *R. padi* would migrate to the northeast and north of Yuanyang county on 7 June 2019 and 28 May 2020, respectively (**Figures 4E, F**).

### Specific aphid facultative symbiont detection

**Figure 5:**
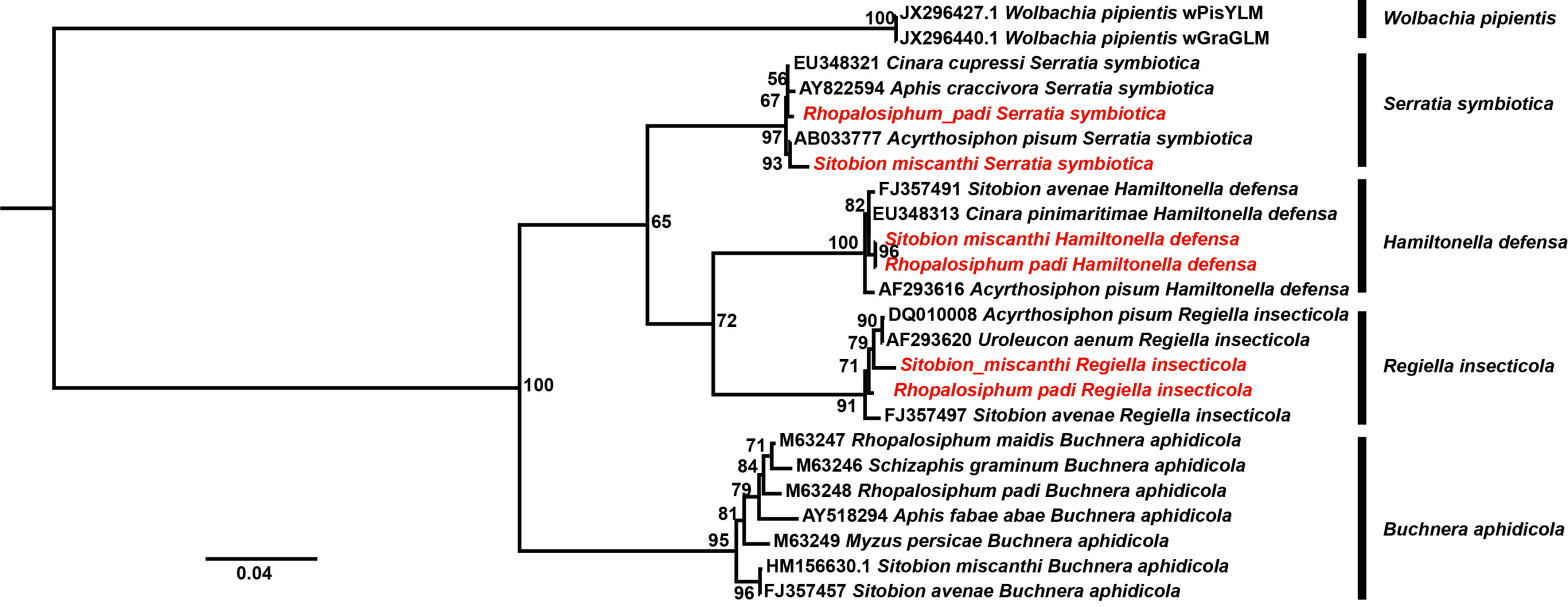
The phylogenetic analysis of aphid bacterial symbionts. The strains identified herein were highlighted with red.

In this study, a total of 237 and 350 samples of *S. miscanthi* and *R. padi* were subjected to the specific bacterial symbiont detections, respectively (**Table 1**). *S. symbiotica*, *H. defensa*, and *R. insercticola* were detected in the aphids; however, no infection of *Wolbachia* was identified. In *S. miscanthi*, infection rates of *S. symbiotica* and *H. defensa* were 12.23% (29/237) and 1.27% (3/237), respectively. On the other side, in *R. padi*, infection rates of *S. symbiotica* and *H. defensa* were 7.71% (27/350) and 1.42% (5/350), respectively. The infection rates of *R. insercticola* in *S. miscanthi* and *R. padi* were 2.95% (7/237) and 4.29 (15/350), respectively. However, between *S. miscanthi* and *R. padi*, no significant difference was observed in the infections of *S. symbiotica* (*χ* ^2^ = 2.28, *p* = 0.13), *H. defensa* (*χ* ^2^ = 0.027, *p* = 0.86), and *R. insercticola* (*χ* ^2^ = 0.69, *p* = 0.40). In the phylogenetic tree (**Figure 5**), the monophyly of *S. symbiotica*, *H. defensa*, and *R. insercticola* was robustly supported. In these symbiont clades, the strains identified in *S. miscanthi* and *R. padi* shared close relationships with that isolated in other aphid species, which further verified their infections.

**Table 1:** Detection of bacterial symbionts in *Sitobion miscanthi* and *Rhopalosiphum padi* with specific primers.

Year	Number of aphids^a^	*Serratia symbiotica* ^b^	*Hamiltonella defensa* ^c^	*Regiella insercticola* ^d^	*Wolbachia* ^e^
2018	60/150	2/8	-/-	-/5	-/-
2019	90/100	15/10	3/-	2/3	-/-
2020	87/100	12/9	-/5	5/7	-/-

a, The number of *Sitobion miscanthi/Rhopalosiphum padi* used in the detection. b–e, The number of positive samples of *Sitobion miscanthi/Rhopalosiphum padi* in the detection. -, No positive sample was detected in the aphids.

### Analyses of 16S rRNA amplicon sequencing

**Figure 6:**
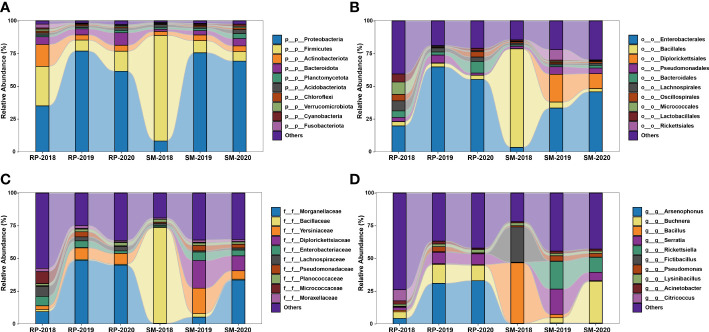
Stacked bar plot showing the relative abundance of the top 10 bacterial taxa identified in *Sitobion miscanthi* and *Rhopalosiphum padi* samples at four taxonomic levels. **(A)**, phylum; **(B)**, order; **(C)**, family; and **(D)**, genus.

In this study, a total of 3,874,860 raw reads (average 129,162 reads per sample) were yielded in the 16S rRNA V3–V4 amplicon sequencing. After trimming, denoising, and chimera removal, 8,518 representative ASVs were obtained, and their taxonomic positions were identified against the SILVA database. The relative abundance of the top 10 bacterial taxa among the samples was revealed at four taxonomic levels (**Figure 6**). The results showed that compositions of bacterial community harbored in the *S. miscanthi* 2018 population was different from that of the remaining aphid populations. For instance, at the phylum level, in the 2018 *S. miscanthi* population, Firmicutes was dominant with 79.60% relative abundance and Proteobacteria was the second main taxon with 8.46% relative abundance. However, in the remaining aphid populations, Proteobacteria was dominant with a relative abundance ranging from 34.49% in the 2018 *R. padi* population to 78.47% in the 2019 *R. padi* population. A similar divergence was also observed at the order and family level. At the genus level, *Buchnera* was identified in all samples with an average relative abundance of 11.26%. Furthermore, two aphid facultative symbionts belonging to *Serratia* and *Rickettsiella* and three common insect bacterial symbionts, *Arsenophonus*, *Rickettsia*, and *Wolbachia*, were identified. In *S. miscanthi*, *Serratia* was the dominant aphid facultative symbiont with an average abundance of 8.41%. On the other side, *Arsenophonus* was the dominant aphid facultative symbiont in *R. padi* with an average abundance of 23.30%. In *S. miscanthi*, the average abundance of *Rickettsiella* was 10.11% much higher than that of *R. padi*, which had only 0.13% average abundance. *Rickettsia* was mainly identified in the 2019 *S. miscanthi* population with a 9.49% abundance. *Wolbachia* was only identified in the 2019 *R. padi* population with a 0.05% abundance. The results of biomarker discovery indicated that *Arsenophonus* was the most differentially abundant bacterial taxon in *R. padi* (**Figure S1**). Additionally, within *S. miscanthi* and *R. padi*, the abundance of *Rickettsiella*, *Arsenophonus*, and *Serratia* was varied among the years. Their lowest relative abundance was observed in the 2018 aphid populations (**Figure S2**). In *S. miscanthi*, the relative abundance of *Rickettsiella* in the 2018 and 2019 aphid populations was significantly different. A similar result was also observed in *Serratia* (**Figure S2A**). In *R. padi*, the relative abundance of *Arsenophonus* in the 2018 aphid population was significantly different from that in the 2019 and 2020 aphid populations (**Figure S2B**).

In this study, the alpha diversity among the samples was varied (**Figure S3A**). Furthermore, it was found that *R. padi* had a relatively higher richness and evenness than *S. miscanthi* (**Figure S3B**). In beta diversity analyses, both PCoA and NMDS analyses indicated that the bacterial community of the 2018 *S. miscanthi* population was distinct from the other samples (**Figure S4**). The samples of *S. miscanthi* trapped in 2019 and 2020 had similar bacterial communities. In *R. padi*, the bacterial communities were separated by years in PCoA. However, in the NMDS analysis, bacterial communities of the *R. padi* samples trapped in 2018 and 2020 and that of *S. miscanthi* samples trapped in 2019 and 2020 were not well separated.

## Discussion

### Population dynamics of wheat migrant wheat aphids

In this study, the amount of migration wheat aphids varied among years. Previous studies indicated that temperature and humidity are the key environmental factors influencing aphid flights (Parry, 2013). Herein, we analyzed the underlying influences of temperature and humidity on the wheat aphid migrations with two types of meteorological data. The results showed that the difference in relative humidity among the years was significant, indicating that relative humidity probably influenced the wheat aphid flight. We also found that the migrating populations of the three wheat aphids varied from each other. *R. padi* was the most abundant among the identified migrating aphids, followed by *S. miscanthi*, and *S. graminum* was the least abundant. This result was consistent with the findings of our aphid surveying in the wheat field (data not shown). In Yuanyang country, *R. padi* was the dominant wheat aphid species, particularly in the late wheat development stages characterized by higher temperatures. Previous studies demonstrated that *R. padi* performed better in high-temperature habitats (Asin and Pons, 2001) and showed greater ecological plasticity when exposed to harsh environments (Zhu et al., 2021). We hypothesize that the *R. padi* population would proliferate faster with a temperature increase than other wheat aphids; thus, more winged *R. padi* would be induced due to the low host plant quality and crowding.

### Migration patterns and simulated trajectories of wheat aphids

A previous study indicated that in Chinese northern winter wheat plant regions, aphids would probably migrate into wheat fields during the wheat heading to flowering stages, and then migrate out in the wheat milk-ripe stage (Li et al., 2014a). In the Yuanyang country, the periods of wheat heading to flowering stages ranged from around late April to early May, and the milk-ripe stage of wheat ranged from around late May to early June. Hence, in this study, we roughly used the half of May as the threshold to distinguish the types of aphid migrations; those that occurred earlier were categorized as immigrations, while those that occurred later were categorized as emigrations. Although alate aphids will undertake appetitive flight in short distances (Loxdale et al., 1993), long-distance migrations are also observed in various aphid species (Kring, 1972). This study found that *R. padi* and *S. miscanthi* had different migration patterns. Specifically, two migration peaks were observed in *R. padi*, identified as immigrations and emigrations. However, in *S. miscanthi*, the migration patterns varied over the years. Only immigrations were observed in 2018, whereas only emigrations were observed in 2019. In 2020, two adjacent migration peaks were uncovered. These results suggest that the field population of *S. miscanthi* in Yuanyang county in 2018 likely included the migrated aphids, but few *S. miscanthi* migrated out in late June. However, in 2019, most *S. miscanthi* field specimens might have been overwintering individuals that migrated out in late May.

Wind speed and direction are the key factors influencing the initiation, path, speed, distance, and duration of aphid flight (Parry, 2013). HYSPLIT is widely used to simulate the migration trajectories of insects, such as *Helicoverpa armigera* (Feng et al., 2009), *Spodoptera frugiperda* (Westbrook et al., 2016), *Pantala flavescens* (Cao et al., 2018), and *Anopheles* mosquitos (Huestis et al., 2019). Herein, HYSPLIT was used to simulate the trajectories of *R. padi* and *S. miscanthi* on their peak migration days. The results indicated that the immigrations typically originated from the south, whereas the north was the primary destination of the emigrations. These findings were consistent with the previous conclusions that wheat aphid migration trajectories in Chinese wheat fields were typically from south to north in line with the monsoons (Li et al., 2014a). However, there were also some exceptions. In 2018, it was predicted that the *S. miscanthi* would migrate from the west, whereas the predicted direction of the *R. padi* in emigrations was south.

### Bacterial composition of wheat aphids

Previously, using specific PCR, *H. defensa* and *R. insercticola* have been detected in *S. miscanthi* (Li et al., 2014b). Recently, the bacterial communities of *S. miscanthi* are investigated with 16S amplicon sequencing (Wang et al., 2022a). However, the examined aphids in these studies are collected in wheat fields; the bacterial community of the migrant wheat aphids is less known. In this study, using specific PCR and phylogenetic analysis, the infections of *S. symbiotica*, *H. defensa*, and *R. insercticola* were identified in *S. miscanthi* and *R. padi* migrants. However, no significant difference was observed in their infection rates. Amplicon sequencing indicated that Proteobacteria and Firmicutes were the dominant bacterial taxa in the wheat aphid migrants. Moreover, we identified two aphid facultative symbionts (*S. symbiotica* and *R. viridis*), and three common insect bacterial symbionts (*Arsenophonus*, *Rickettsia*, and *Wolbachia*). *Arsenophonus* has been detected in diverse aphid groups (Jousselin et al., 2012), whereas *R. viridis* has been identified in pea aphid (Tsuchida et al., 2010). To our knowledge, it is the first report of them in *S. miscanthi* and *R. padi.* Furthermore, *Arsenophonus* was identified to be the most differentially abundant bacterial taxon in *R. padi*. Additionally, to uncover the potential interactions between the infections of bacterial symbionts and aphid flight, the relative abundance of *Rickettsiella*, *Arsenophonus*, and *Serratia*, was used in the statistical analyses. In both *S. miscanthi* and *R. padi*, the lowest relative abundance of the bacterial symbionts was observed in the 2018 aphid populations. Furthermore, the specific PCR results revealed that the 2018 populations of *S. miscanthi* and *R. padi* had the lowest rates of bacterial symbiont infection. Since most of the aphid migrants were trapped in 2018, it indicated that aphid bacterial symbionts did not probably promote the wheat aphid migrations. However, this hypothesis needs to be further verified by comparing the aphid flight capabilities of bacterial symbiont infected and uninfected populations.

In conclusion, in this study, we used the data obtained from the Yuanyang suction trap site, uncovered the population dynamics of migrant wheat aphids in the main wheat planting region in China, and simulated their migration trajectories. Furthermore, we revealed the potential roles of climate conditions on the aphid migrations. Additionally, the interactions between wheat aphid migrants and bacteria were investigated with specific PCR and amplicon sequencing.

## Data availability statement

The original contributions presented in the study are publicly available. This data can be found here: NCBI, PRJNA898692.

## Author contributions

TL, YW, and CL conceived the study. TL, GY, QL, and YJ drafted the manuscript. DK, ZF, RL, and GZ collected and identified the aphid species.TL prepared the figures. TL, YW, and QL revised the manuscript. TL, YW, and CL designed the whole study and revised the manuscript. All authors contributed to the article and approved the submitted version.

## Supplementary material

Supplementary Figure 1 The cladogram and abundance of differential species. **(A)** A cladogram showing the significant differentially abundant taxa in *Sitobion miscanthi* and *Rhopalosiphum padi*. Significant differentially abundant taxa were identified by the Kruskal-Wallis test (p< 0.05). **(B)** The abundance and LDA effect size of differential taxa in *Sitobion miscanthi* and *Rhopalosiphum padi*.

Supplementary Figure 2 Statistic analysis of the relative abundance of *Rickettsiella*, *Arsenophonus*, and *Serratia* among years. **(A)** Analysis performed in *Sitobion miscanthi*. **(B)** Analysis performed in *Rhopalosiphum padi*.

Supplementary Figure 3 The boxplots of alpha diversity indexes. **(A)** The ACE, Chao1, Shannon and Simpson indexes of bacterial communities in the *Sitobion miscanthi* and *Rhopalosiphum padi* populations trapped during 2018-2020. **(B)** The ACE, Chao1, Shannon and Simpson indexes of bacterial communities of the entire *Sitobion miscanthi* and *Rhopalosiphum padi* samples. SM, *Sitobion miscanthi*; RR, *Rhopalosiphum padi*.

Supplementary Figure 4 The results of beta diversity analyses. **(A)** The PCoA analyses among the *Sitobion miscanthi* and *Rhopalosiphum padi* samples. **(B)** The NMDS analyses among the *Sitobion miscanthi* and *Rhopalosiphum padi* samples.
