# Supplementary figures and images for: Population dynamics of migrant wheat aphids in China’s main wheat production region and their interactions with bacterial symbionts
Source: Front Plant Sci. 2023 Feb 9;14:1103236. doi: 10.3389/fpls.2023.1103236 (PMC9947703; doi:10.3389/fpls.2023.1103236)

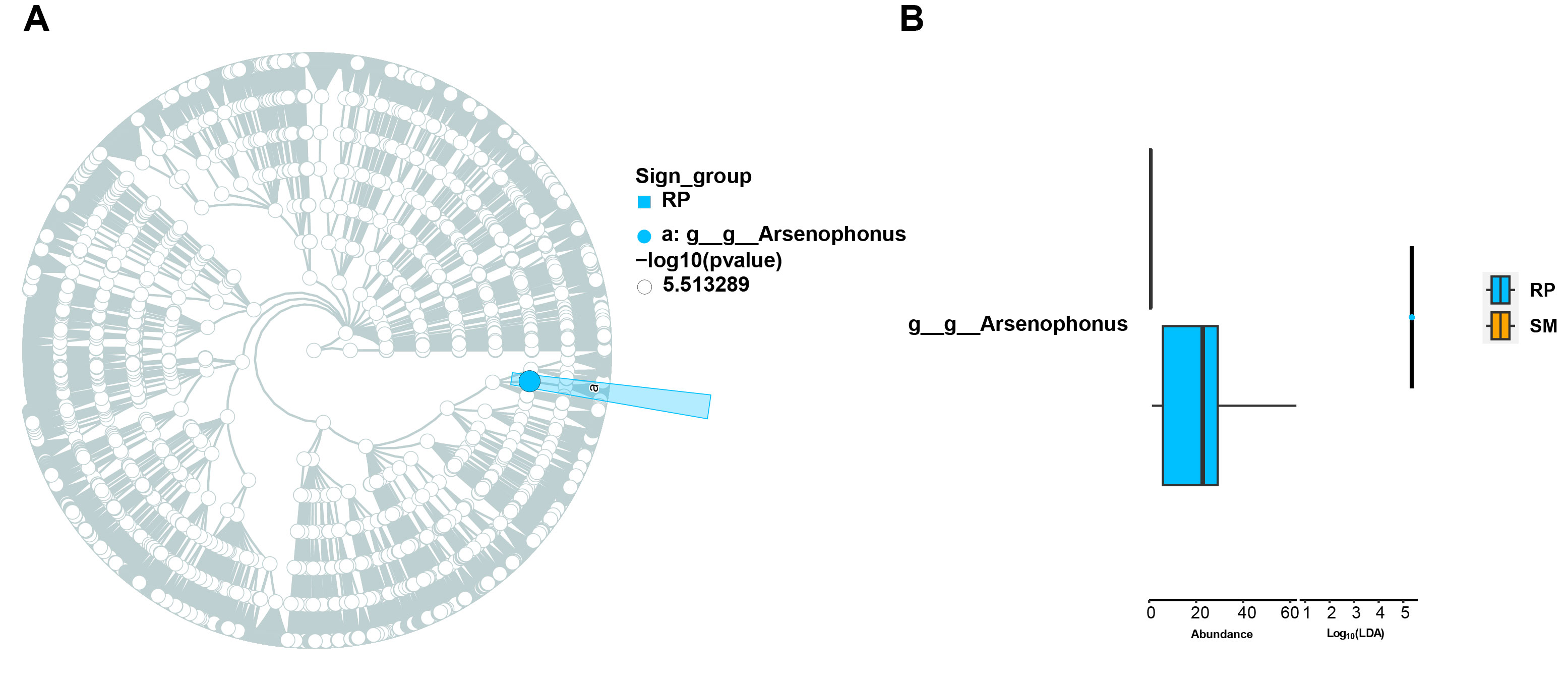

Supplement: Supplementary Figure 1 — The cladogram and abundance of differential species. (A) A cladogram showing the significant differentially abundant taxa in Sitobion miscanthi and Rhopalosiphum padi. Significant differentially abundant taxa were identified by the Kruskal-Wallis test (p< 0.05). (B) The abundance and LDA effect size of differential taxa in Sitobion miscanthi and Rhopalosiphum padi. [file Image_1.jpeg]

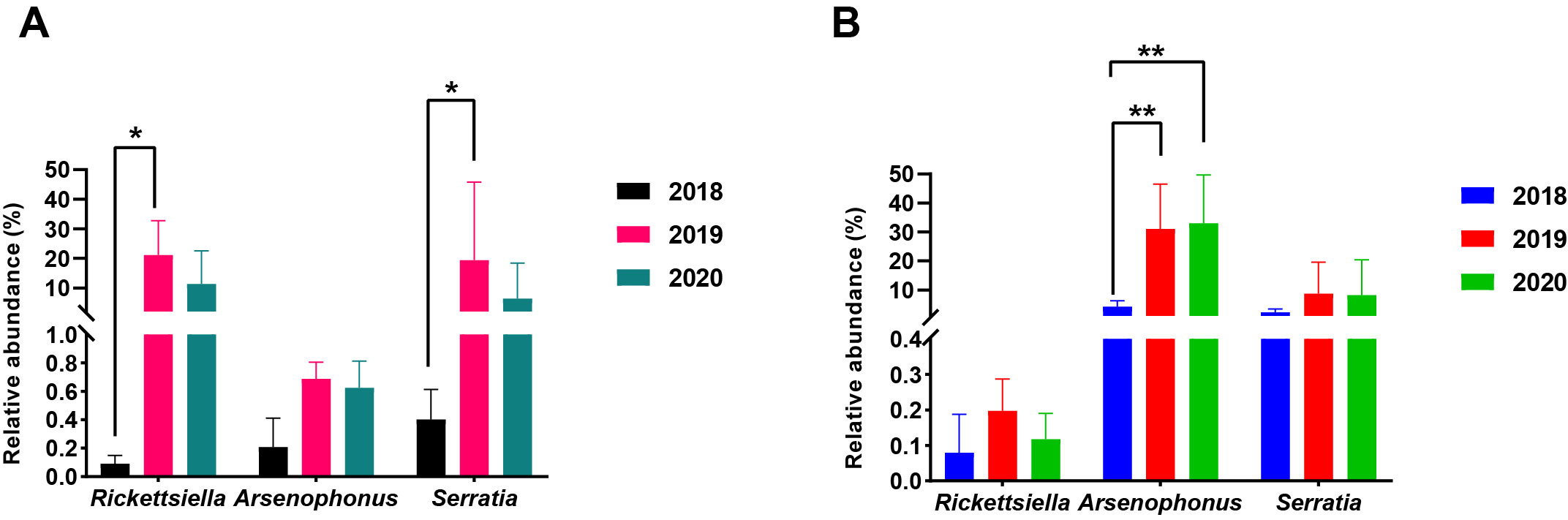

Supplement: Supplementary Figure 2 — Statistic analysis of the relative abundance of Rickettsiella, Arsenophonus, and Serratia among years. (A) Analysis performed in Sitobion miscanthi. (B) Analysis performed in Rhopalosiphum padi. [file Image_2.jpeg]

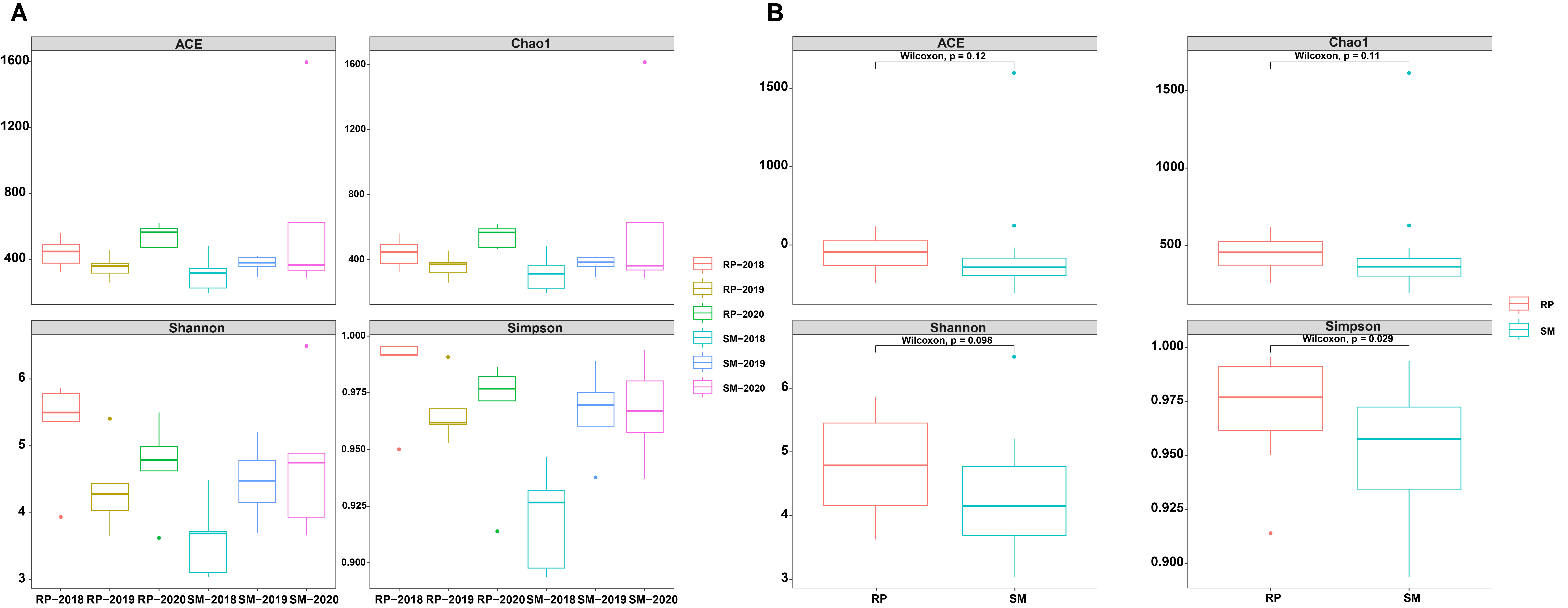

Supplement: Supplementary Figure 3 — The boxplots of alpha diversity indexes. (A) The ACE, Chao1, Shannon and Simpson indexes of bacterial communities in the Sitobion miscanthi and Rhopalosiphum padi populations trapped during 2018-2020. (B) The ACE, Chao1, Shannon and Simpson indexes of bacterial communities of the entire Sitobion miscanthi and Rhopalosiphum padi samples. SM, Sitobion miscanthi; RR, Rhopalosiphum padi. [file Image_3.jpeg]

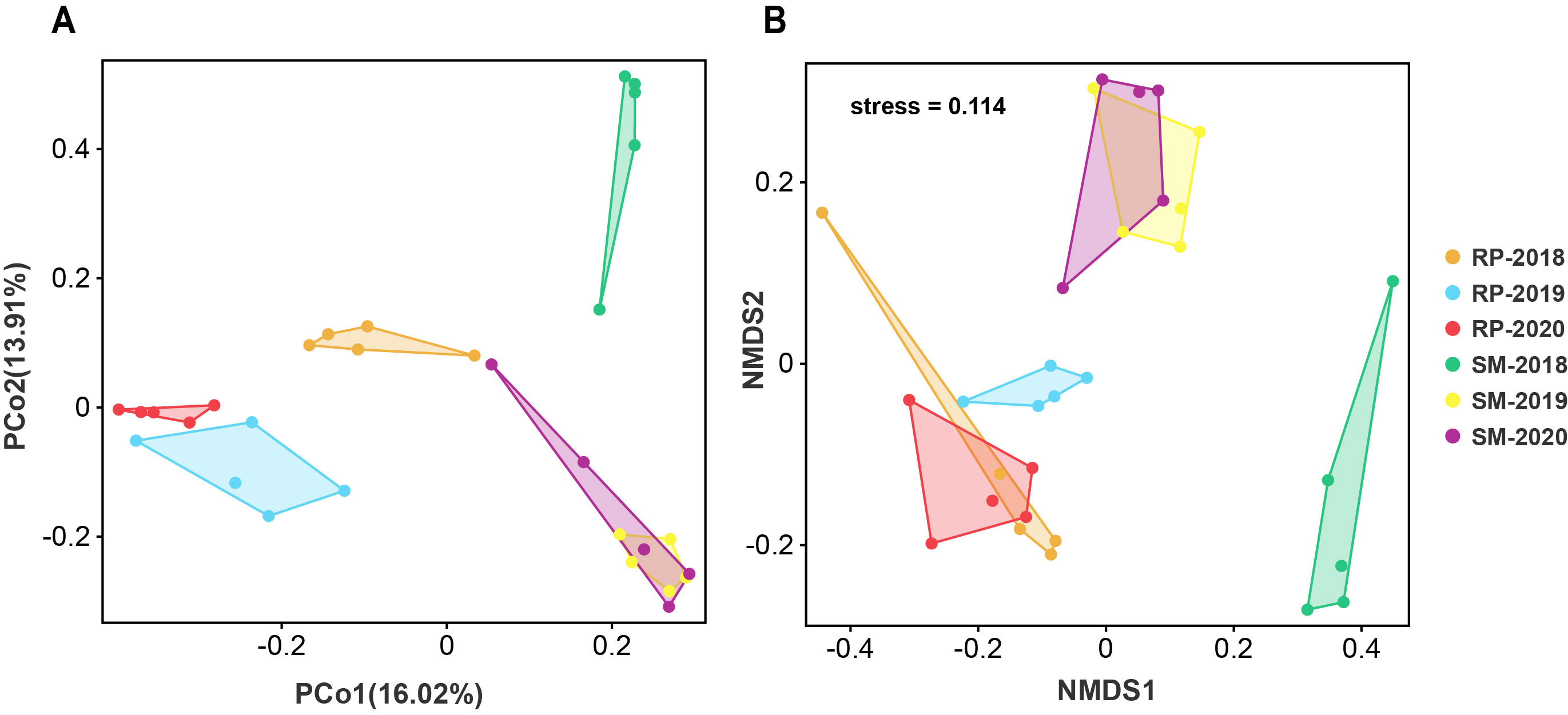

Supplement: Supplementary Figure 4 — The results of beta diversity analyses. (A) The PCoA analyses among the Sitobion miscanthi and Rhopalosiphum padi samples. (B) The NMDS analyses among the Sitobion miscanthi and Rhopalosiphum padi samples. [file Image_4.jpeg]
