# Supplementary material for: Population dynamics of migrant wheat aphids in China’s main wheat production region and their interactions with bacterial symbionts
Source: Front Plant Sci. 2023 Feb 9;14:1103236. doi: 10.3389/fpls.2023.1103236 (PMC9947703; doi:10.3389/fpls.2023.1103236)
Supplement: Supplementary file 5 [file Table_1.docx]

**Table S1. Specific primers used in this study.**

| Organism | Gene | Primer name | Fragment length | Primer sequences | Reference |
| --- | --- | --- | --- | --- | --- |
| *Serratia symbiotica* | 16S rRNA | 16sa1 | 470bp | agagtttgatcmtggctcag | (Fukatsu & Nikoh, 1998) |
|  |  | PASScmp |  | GCAATGTCTTATTAACACAT | (Fukatsu & Nikoh, 2000) |
| *Hamiltonella defensa* | 16S rRNA | T99F | 1400bp | AGTGAGCGCAGTTTACTGAG | (Sandstrom et al., 2001) |
|  |  | 16sb1 |  | tacggytaccttgttacgactt | (Fukatsu & Nikoh, 1998) |
| *Regiella insecticola* | 16S rRNA | 10F | 400bp | AGTTTGATCATGGCTCAGATTG | (Henry et al., 2013) |
|  |  | TO419R |  | GGTAACGTCAATCGATAAGCA | (Henry et al., 2013) |
| *Wolbachia* | 16S rRNA | wol-16s-F | 1000bp | ttgtagcctgctatggtataact | (O'Neill et al., 1992) |
|  |  | wol-16s-R |  | gaataggtatratttycatgt | (O'Neill et al., 1992) |

References

Fukatsu, T. and N. Nikoh (1998). Two intracellular symbiotic bacteria from the mulberry psyllid *Anomoneura mori* (Insecta, Homoptera). *Appl. Environ. Microbiol.* 64(10): 3599-3606. doi: 10.1128/aem.64.10.3599-3606.1998

Fukatsu, T. and N. Nikoh (2000). Endosymbiotic microbiota of the bamboo pseudococcid Antonina crawii (Insecta, Homoptera). *Appl. Environ. Microbiol.* 66(2): 643-650. doi: 10.1128/aem.66.2.643-650.2000

Henry, L. M., J. Peccoud, J. C. Simon, J. D. Hadfield, M. J. Maiden, J. Ferrari and H. C. Godfray (2013). Horizontally Transmitted Symbionts and Host Colonization of Ecological Niches. *Curr. Biol.* 23: 1713-1717 doi: 10.1016/j.cub.2013.07.029

Sandstrom, J. P., J. A. Russell, J. P. White and N. A. Moran (2001). Independent origins and horizontal transfer of bacterial symbionts of aphids. *Mol. Ecol.* 10(1): 217-228. doi: 10.1046/j.1365-294x.2001.01189.x.

O'Neill, S. L., R. Giordano, A. M. E. Colbert, T. L. Karr and H. M. Robertson (1992). 16S rRNA Phylogenetic Analysis of the Bacterial Endosymbionts Associated with Cytoplasmic Incompatibility in Insects. *PNAS* 89(7): 2699-2702. doi: 10.1073/pnas.89.7.2699
